# Sex-specific differences in abscopal responses to combined radiotherapy and immune checkpoint inhibition–insights from a multicenter study

**DOI:** 10.3389/fimmu.2025.1699362

**Published:** 2026-02-02

**Authors:** Maike Trommer, Alexander Rühle, Felix Ehret, Allison Lamrani, Charlotte Schmitter, Justus Kaufmann, Matthias Mäurer, Georg Wurschi, Ping Jiang, Andrea Baehr, Annika Hardt, Raphael Bodensohn, Lukas Käsmann, Maria Waltenberger, Eleni Gkika, Davide Scafa, Julian P. Layer, Esther G.C. Troost, Sally A. Elkhamisy, Danny Jazmati, Ilinca Popp, Sebastian Neppl, Anna Hagemeier, Angela Besserer, Simone Ferdinandus

**Affiliations:** 1 Department of Radiation Oncology, Faculty of Medicine and University Hospital Bonn, Bonn, Germany; 2 Department of Radiation Oncology, Medical Center, Faculty of Medicine, University of Freiburg, Freiburg, Germany; 3 Department of Radiation Oncology, University of Leipzig Medical Center, Leipzig, Germany; 4 Department of Radiation Oncology, Charité-Universitätsmedizin Berlin, Corporate Member of Freie Universität Berlin and Humboldt-Universität zu Berlin, Berlin, Germany; 5 German Cancer Consortium (DKTK), partner site Berlin, a partnership between DKFZ and Charité – Universitätsmedizin, Berlin, Germany; 6 Department of Radiation Oncology, Universitätsklinikum Erlangen, Friedrich-Alexander-Universität Erlangen-Nürnberg, Erlangen, Germany; 7 Comprehensive Cancer Center Erlangen-Erlangen-Metropolregion Nürnberg (EMN), Erlangen, Germany; 8 Department of Radiation Oncology, University Medical Center of the Johannes Gutenberg University, Mainz, Germany; 9 Department of Radiotherapy and Radiation Oncology, Jena University Hospital, Jena, Germany; 10 Clinician Scientist Program OrganAge, Jena University Hospital, Jena, Germany; 11 Clinician Scientist Program, Interdisciplinary Center for Clinical Research (IZKF), Jena University Hospital, Jena, Germany; 12 Clinic for Radiation Oncology and Radiotherapy, Lüdenscheid Clinic, Lüdenscheid, Germany; 13 Department of Radiation Oncology, University Medical Hospital, Hamburg-Eppendorf, Hamburg, Germany; 14 Department of Radiotherapy and Radiation Oncology, Outpatient Center of the University Medical Hospital Hamburg-Eppendorf, Hamburg,, Germany; 15 Department of Radiation Oncology, University Hospital, Ludwig-Maximilians-Universität (LMU) Munich, Munich, Germany; 16 Department of Radiation Oncology, University Hospital Tübingen, Tübingen, Germany; 17 Department of Radiation Oncology, Hospital of Bolzano (SABES-ASDAA), Bolzano-Bozen, Italy; 18 Teaching Hospital of Paracelsus Medical University Salzburg, Salzburg, Austria; 19 Department of Radiation Oncology, Klinikum rechts der Isar, Technical University of Munich, Munich, Germany; 20 Institute of Experimental Oncology, University Hospital Bonn, Bonn, Germany; 21 OncoRay – National Center for Radiation Research in Oncology, Faculty of Medicine and University Hospital Carl Gustav Carus, Technische Universität Dresden, and Helmholtz-Zentrum Dresden- Rossendorf, Institute of Radiooncology – OncoRay Helmholtz- Zentrum Dresden-Rossendorf, Dresden, Germany; 22 National Center for Tumor Diseases (NCT/UCC), Dresden, Germany; 23 National Center for Tumor Diseases (NCT/UCC), Dresden, Germany; German Cancer Research Center (DKFZ), Heidelberg, Germany, Medizinische Fakultät and University, Hospital Carl Gustav Carus Technische Universität Dresden, Germany; Helmholtz-Zentrum Dresden-Rossendorf (HZDR), Dresden, Germany; 24 University Hospital Dusseldorf, Medical Faculty, Heinrich-Heine-University Dusseldorf, Department of Radiation Oncology, Dusseldorf, Germany; 25 Department of Radiation Oncology, Cyberknife and Radiotherapy, Faculty of Medicine and University Hospital Cologne, Cologne, Germany; 26 Institute of Medical Statistics and Computational Biology, Medical Faculty and University Hospital, University of Cologne, Cologne, Germany; 27 Department of Radiation Oncology, Ernst von Bergmann Hospital Potsdam, Potsdam, Germany

**Keywords:** abscopal effects, sex-specific immune effects, sex-specific radiation oncology, radioimmunotherapy, immune checkpoint inhibition

## Abstract

**Purpose:**

Abscopal effects (AbE) during combined radiotherapy (RT) and immune checkpoint inhibition (ICI) represent a potential mechanism for systemic tumor control, yet sex-specific differences in these responses remain largely unexplored. We investigated sex-associated signals in outcomes of combined RT-ICI in a multicenter cohort. We analyzed the incidence of AbE and survival outcomes with respect to clinical and biomedical markers.

**Methods:**

In this observational multicenter study, patients with metastatic solid tumors receiving RT-ICI and showing at least one non-irradiated lesion (NIL), assessed using iRECIST criteria, were analyzed. Abscopal response (AR) was defined as ≥30% reduction in NIL size, abscopal progression (AP) as ≥20% increase, and abscopal control (AC) as changes within this range.

**Results:**

Among 3,773 screened patients, 142 met the inclusion criteria (62% male, median age 62 years; 38% female, median age 58 years). AR and AC occurred more frequently in females (24% vs. 14%, 35% vs. 31%). While OS showed no significant difference (p=0.81), Cox regression analyses revealed significant associations of a longer ICI-to-RT-interval (males: HR = 0.903 [0.833–0.978], p=0.012; females: HR = 0.748 [0.621–0.900], p=0.002) and a BMI ≥25 kg/m² with survival in both sexes (males: HR = 4.282 [1.473–12.446], p=0.008; females: HR = 4.801 [1.182–19.502], p=0.028 with survival in both sexes). Elevated C-reactive protein (CRP) (≥5 mg/L) showed prognostic significance only in males (HR = 4.764 [1.184–19.170], p=0.028).

**Conclusion:**

Our findings suggest the possibility of sex-specific patterns in AbE occurrence. Additionally, our analyses identified sex-associated prognostic factors, including the importance of ICI-to-RT interval and BMI in both sexes and the male-specific prognostic value of CRP. These observations warrant further research and consideration in designing personalized RT-ICI combination strategies.

## Highlights

- Females showed higher rates of abscopal response (33% vs. 26%) and control (35% vs. 31%) compared with men.
- Longer intervals between ICI and RT were associated with improved survival in both sexes.
- High BMI (≥25 kg/m²) was a negative prognostic factor for both men and women.
- Elevated CRP (≥5 mg/L) was associated with worse survival only in male patients.
- Sex-specific immune responses may influence RT-ICI efficacy, supporting personalized treatment strategies.

## Introduction

The combination of radiation therapy (RT) and immune checkpoint inhibition (ICI) has emerged as a promising therapeutic strategy, particularly notable for its potential to induce abscopal effects (AbE) - an immune-mediated response characterized by tumor regression at sites distant from the irradiated field (1, 2). RT can act as an *in situ* vaccine, triggering immunogenic cell death and release of damage-associated molecular patterns (DAMPs), which can synergize with ICI to enhance systemic antitumor immunity (3, 4). This combination is particularly promising for patients experiencing disease progression under ICI (5, 6), though the determinants of response remain poorly understood.

Sex-based differences in immune responses have been documented primarily in recent years, with females generally exhibiting more robust immune activation compared to males, which, on the one hand, may lead to a higher efficacy of vaccination but, on the other hand, to more frequent adverse reactions (7, 8). This sexual dimorphism extends to cancer immunotherapy, where some studies have demonstrated immunotherapy being more effective in males compared to females, which also translates into a greater average survival benefit from immunotherapy for male patients (9, 10).

Evidence specifically addressing sex-specific differences in AbE frequency and impact on clinical outcomes remains limited. Recent findings collectively underscore the growing recognition that sex represents a crucial biological variable in determining immunotherapy efficacy, suggesting that sex-specific considerations should be integrated into treatment planning and response assessment.

We have conducted a nationwide multicenter retrospective assessment of the occurrence and patterns of AbE in a real-world cohort of metastasized patients – the so-called ARTIC (Abscopal effects in metastasized cancer patients treated with RadioTherapy and Immune Checkpoint inhibition) study (11), in which patients received RT of any type after progressing during ICI treatment. In this secondary analysis, we elucidate differences in AbE occurrence and their prognostic implications with regard to biological sex, while exploring potential sex-associated signals. By investigating the complex interplay between sex, immune response, and inflammatory markers, we seek to advance the development of more personalized treatment approaches in radiation oncology and, eventually, improve patient outcomes through sex-specific stratification strategies.

## Methods

### Study design and patient data

This retrospective, non-interventional, multicenter observational study is based on a real-world patient cohort. The study design and its objective were previously published (11). The study design is depicted in **Supplementary Figure 1**. The study population consisted of patients with metastasized solid tumors of any origin treated between 2015 to 2021 at participating centers with ICI and concomitant RT meeting the inclusion criteria. Statistical case number estimation required approximately 70 patients to be included with an accuracy of 10% (confidence interval, CI 10-30%) for an estimated AbE rate of 20%. To achieve our target of 70 patients, we planned to screen at least 460 patients. The initial target of 70 analyzable patients reflected a feasibility estimate; all centers subsequently screened consecutive ICI+RT cases over the finalized window using identical criteria.

Centers provided de-identified exports with unique local IDs. Duplicates were removed by retaining the first eligible course per ID. After passing the initial database pre-screening, patients were assessed for inclusion and exclusion criteria. The inclusion criteria for this study required patients to have received local RT for one or more metastases and/or the primary tumor, with any type of external beam RT techniques and brachytherapy. We grouped RT types according to the administered single dose in hyperfractionated (< 1,8 Gy), conventionally fractionated (1,8 - 2,2 Gy), hypofractionated (2,3–4 Gy), and ultrahypofractionated/stereotactic (≥ 5 Gy) RT (12) (13),. Any type of ICI was allowed. To assess AbE, patients had to have at least one lesion outside the 10% isodose, with at least two radiological images of non-irradiated lesions (NIL) before RT initiation and at least one follow-up image after RT completion (see **Supplementary Figure 1**). Exclusion criteria included inadequate imaging, tumor response to ICI alone before the initiation of RT, or initiation of another systemic therapy during the analyzed period. Anthropometric data, as well as blood biomarkers [C-reactive protein (CRP)] and details of ICI treatment, were collected. For RT data, localization, fractionation schemes, and RT dose were collected. RT localization was further divided into two categories, brain” and “other”, to account for the distinct effects of the blood-brain barrier. Data compilation and clearing were performed at the principal investigation center (MT and SF).

### Lesion-based analysis

For assessment of NILs, we identified up to 5 of the largest lesions, which were radiologically confirmed metastatic lesions and visible in the first two scans (pre-RT). A minimum of 3 images was required (see **Supplementary Figure 1**). NILs were measured to their largest extent (lymph nodes in their short axis diameter) and NIL assessment was performed according to iRECIST criteria (14, 15): Diameter of lesions showing ≥30% decrease in size: “abscopal response” (AR), ≥20% increase in size: “abscopal progression” (AP), between <30% decrease and <20% increase in size: “abscopal control” (AC). Patients were further grouped as patients with AR in all lesions, patients with at least one lesion with AR while other lesions were stable (≥ 1 AR), patients with all lesions stable (AC), patients with progressive disease in all lesions (AP), and mixed response (other). To simplify these categories, we combined the patients with “AR”, ≥ 1 AR”, and “AC” into the “abscopal benefit (AB)” group. The “no abscopal benefit (no AB)” group consisted of patients with AP and mixed responses. For a visual representation of this stratification, please refer to **Supplementary Figure 2**.

### Statistical analysis

Descriptive analyses were conducted to summarize the study population. Categorical variables are presented as absolute and relative frequencies, while continuous variables are reported using means with standard deviations (SD) and medians with interquartile ranges (IQR). Kaplan-Meier survival curves were created to evaluate overall survival (OS) and progression-free survival (PFS), stratified by the presence or absence of AB. Cox regression analysis was performed to identify factors influencing overall survival. Variables were selected based on clinical relevance, then categorical variables with sparse categories were grouped together in a meaningful way. This resulted in the following multivariable Cox regression model with the variables age at diagnosis, time between ICI and RT, RT location categorized as brain and other, RT dose type categorized as stereotactic, hypofractionated and other, BMI categorized as overweight and no overweight, prior RT (Yes/No), smoking status (current smoker/former or never smoker) and CRP before RT categorized as ≥5/<5 mg/L. The proportional hazards assumption was confirmed using Schoenfeld residuals and the global Schoenfeld test. Comparison tests (log-rank tests) were only calculated if the assumption was fulfilled. Patients were stratified by sex, and all relevant baseline characteristics, treatment parameters, and outcome measures were analyzed separately for each group. The Cox regression model was applied separately to identify sex-specific influencing factors while maintaining model stability. Cox regression analyses were conducted on complete cases; because CRP values were missing in approximately 30% of patients, sensitivity analyses were performed excluding this variable to assess potential bias. AR was not included as a covariate in the Cox regression models, as the occurrence of AR represents a post-radiotherapy event. Statistical significance was defined as a p-value of ≤ 0.05, though all p-values were exploratory and not adjusted for multiple testing. The analyses were conducted using R version 4.4.0 (16).

The study was registered with the German working group for radiation oncology (Arbeitsgemeinschaft Radiologische Onkologie (ARO), trial number: ARO 2022-07) and DRKS (Deutsches Register für Klinische Studien, DRKS00032390). The local institutional review board (Ethics Committee of the University of Cologne) approved the study (22-1230-retro).

## Results

### Patient characteristics

A total of 3773 cases were screened across 12 contributing centers, resulting in the inclusion of N = 142 patients. Of these, 38.0% (n=54) were female and 61.9% (n=88) male. The mean age at diagnosis was 56.5 ± 16.1 years for female patients and 61.4 ± 11.9 years for male patients. The mean BMI was 23.8 ± 5.07 kg/m² in females and 25.3 ± 4.37 kg/m² in males. Among female patients, 51.9% (n=28) received Nivolumab as their ICI therapy, 42.6% (n=23) were treated with Pembrolizumab, and 1.9% (n=1) with Atezolizumab. In male patients, Nivolumab was used in 45.5% (n=40) and Pembrolizumab in 44.3% (n=39). Cemiplimab, Durvalumab, Atezolizumab, and Avelumab were each administered in 2.3% (n=2) of male patients. Prior RT had not been administered in 35.2% (n=19) of female and 43.2% (n=38) of male patients. The most common cancer types in females were melanoma with 38.9% (n=21) and non-small cell lung cancer (NSCLC) with 38.9% (n=21). In males, NSCLC with 39.8% (n=35) was the most frequent tumor type, followed by melanoma with 30.7% (n=27). For a summary of patient characteristics, see **Table 1**.

**Table 1:** Patient characteristics.

	Male	Female	Total
	(N = 88)	(N = 54)	(N = 142)
Age at diagnosis (y)			
Median [Q1;Q3]	62.0 [55.8;70.0]	58.0 [48.0;67.8]	61.0 [52.0;69.8]
Mean ± SD	61.4 ± 11.9	56.5 ± 16.1	59.6 ± 13.8
Missing	4 (4.5%)	4 (7.4%)	8 (5.6%)
Cancer type			
Melanoma	27 (30.7%)	21 (38.9%)	48 (33.8%)
NSCLC	35 (39.8%)	21 (38.9%)	56 (39.4%)
Renal Cell cancer	8 (9.1%)	4 (7.4%)	12 (8.5%)
Other (frequencies <5%)	18 (20.5%)	8 (14.8%)	26 (18.3%)
ICI end to RT start (m)			
Median [Q1;Q3]	2.50 [1.00;8.00]	2.00 [0;8.25]	2.00 [1.00;8.00]
Missing	6 (6.8%)	6 (11.1%)	12 (8.5%)
ICI type			
Pembrolizumab	39 (44.3%)	23 (42.6%)	62 (43.7%)
Nivolumab	40 (45.5%)	28 (51.9%)	68 (47.9%)
Cemiplimab	2 (2.3%)	0 (0%)	2 (1.4%)
Durvalumab	2 (2.3%)	0 (0%)	2 (1.4%)
Atezolizumab	2 (2.3%)	1 (1.9%)	3 (2.1%)
Other	3 (3.4%)	0 (0%)	3 (2.1%)
Missing	0 (0%)	2 (3.7%)	2 (1.4%)
RT localization			
brain	26 (29.5%)	14 (25.9%)	40 (28.2%)
other	62 (70.5%)	40 (74.1%)	102 (71.8%)
RT type (grouped)			
ultrahypofractionated/stereotactic	30 (34.1%)	21 (38.9%)	51 (35.9%)
hypofractionated	47 (53.4%)	25 (46.3%)	72 (50.7%)
other	11 (12.5%)	8 (14.8%)	19 (13.4%)
BMI at RT start (kg/m^2^)			
Mean ± SD	25.3 ± 4.37	23.8 ± 5.07	24.7 ± 4.71
Missing	10 (11.4%)	2 (3.7%)	12 (8.5%)
Prior RT			
No	38 (43.2%)	19 (35.2%)	57 (40.1%)
Yes	46 (52.3%)	34 (63.0%)	80 (56.3%)
Missing	4 (4.5%)	1 (1.9%)	5 (3.5%)
Smoking status			
never smoker	31 (35.2%)	25 (46.3%)	56 (39.4%)
former smoker	29 (33.0%)	15 (27.8%)	44 (31.0%)
current smoker	16 (18.2%)	7 (13.0%)	23 (16.2%)
Missing	12 (13.6%)	7 (13.0%)	19 (13.4%)
CRP at RT start (mg/L)			
Mean ± SD	20.8 ± 35.7	26.1 ± 34.2	22.9 ± 35.0
Missing	28 (31.8%)	14 (25.9%)	42 (29.6%)
Response 3m follow-up			
Complete Remission	2 (2.3%)	1 (1.9%)	3 (2.1%)
Partial Remission	16 (18.2%)	13 (24.1%)	29 (20.4%)
Stable Disease	18 (20.5%)	6 (11.1%)	24 (16.9%)
Progressive Disease	29 (33.0%)	22 (40.7%)	51 (35.9%)
Mixed Response	23 (26.1%)	10 (18.5%)	33 (23.2%)
Pseudoprogression	0 (0%)	0 (0%)	0 (0%)
Missing	0 (0%)	2 (3.7%)	2 (1.4%)
Abscopal effect			
AR	12 (13.6%)	13 (24.1%)	25 (17.6%)
AC	27 (30.7%)	19 (35.2%)	46 (32.4%)
≥1 AR	11 (12.5%)	5 (9.3%)	16 (11.3%)
AP	8 (9.1%)	1 (1.9%)	9 (6.3%)
Other	30 (34.1%)	16 (29.6%)	46 (32.4%)
Abscopal benefit			
AR, AC & ≥1 AR (yes)	50 (56.8%)	37 (68.5%)	87 (61.3%)
AP & other (no)	38 (43.2%)	17 (31.5%)	55 (38.7%)

AR, Abscopal Response; AC, Abscopal Control; AP, Abscopal Progression; AB, Abscopal Benefit; NSCLC, non-small-cell lung cancer; MM, malignant melanoma; RCC, renal cell cancer; ICI, immune checkpoint inhibitor, other ICI type: Avelumab (n=2), Tislelizumab (n=1); RT; radiotherapy; BMI, body mass index; CRP, C-reactive protein; Gy; Gray; EQD2, equivalent dose in 2 Gy fractions, other RT type: conventionally fractionated (n=18), hyperfractionated (n=1, female); m; months; y, years.

### Radiotherapy

Among female patients, 79.6% (n=43) received a single course of RT, while 20.4% (n=11) underwent two concurrent courses. Similarly, 86.4% (n=76) of male patients received one RT course, whereas 13.6% (n=12) were treated with two. Moderately hypofractionated RT was the most common fractionation scheme in both groups, applied in 46.3% (n=25) of female and 53.4% (n=47) of male patients, followed by ultrahypofractionated/stereotactic RT (38.9% (n=21) and 34.1% (n=30), respectively). The most frequently targeted area in female patients was the lung (25.9%, n=14), followed closely by the brain (22.2%, n=12) and bone (20.4%, n=11). Among male patients, RT was most often applied to the brain (28.4%, n=25), compared to the bone (20.5%, n=18), lung (17.0%, n=15), and lymphatic system (17.0%, n=15). The mean total physical dose delivered (± SD) was 33.0 ± 13.9 Gy in female patients and 37.3 ± 13.8 Gy in male patients. The mean single dose of RT was 6.53 ± 6.10 Gy for females and 6.01 ± 5.85 Gy for males.

### Patient-based and lesion-based analysis

Among female patients, 24.1% (n=13) exhibited AR, while 35.2% (n=19) showed AC. Furthermore, 9.3% (n=5) were classified as ≥1AR, whereas only 1.9% (n=1) fell into the AP category. 29.6% (n=16) were grouped as Other due to mixed responses. Overall, 68.5% (n=37) of female patients showed AB (AR, AC & ≥1AR), while 31.5% (n=17) did not (AP & Other). Among male patients, the proportions of AR and AC were lower, while AP was more frequent. 13.6% (n=12) of males had AR, and 30.7% (n=27) had AC. 12.5% (n=11) were classified as ≥1AR, whereas 9.1% (n=8) were in the AP group. 34.1% (n=30) exhibited mixed responses and were categorized as Other. In total, 56.8% (n=50) of male patients showed AB, while 43.2% (n=38) did not. The detailed patient-based descriptive analysis is demonstrated in **Table 1**. In the lesion-based analysis, 26.4% (32/121) of NILs in female patients showed a ≥30% decrease. Among male patients, the AR rate in NILs was 18.9% (42/222) of lesions. The overall AR rate was 21.6% (74/343) (data not shown).

### Survival analysis (Kaplan Meier and cox regression)

**Figure 1:**
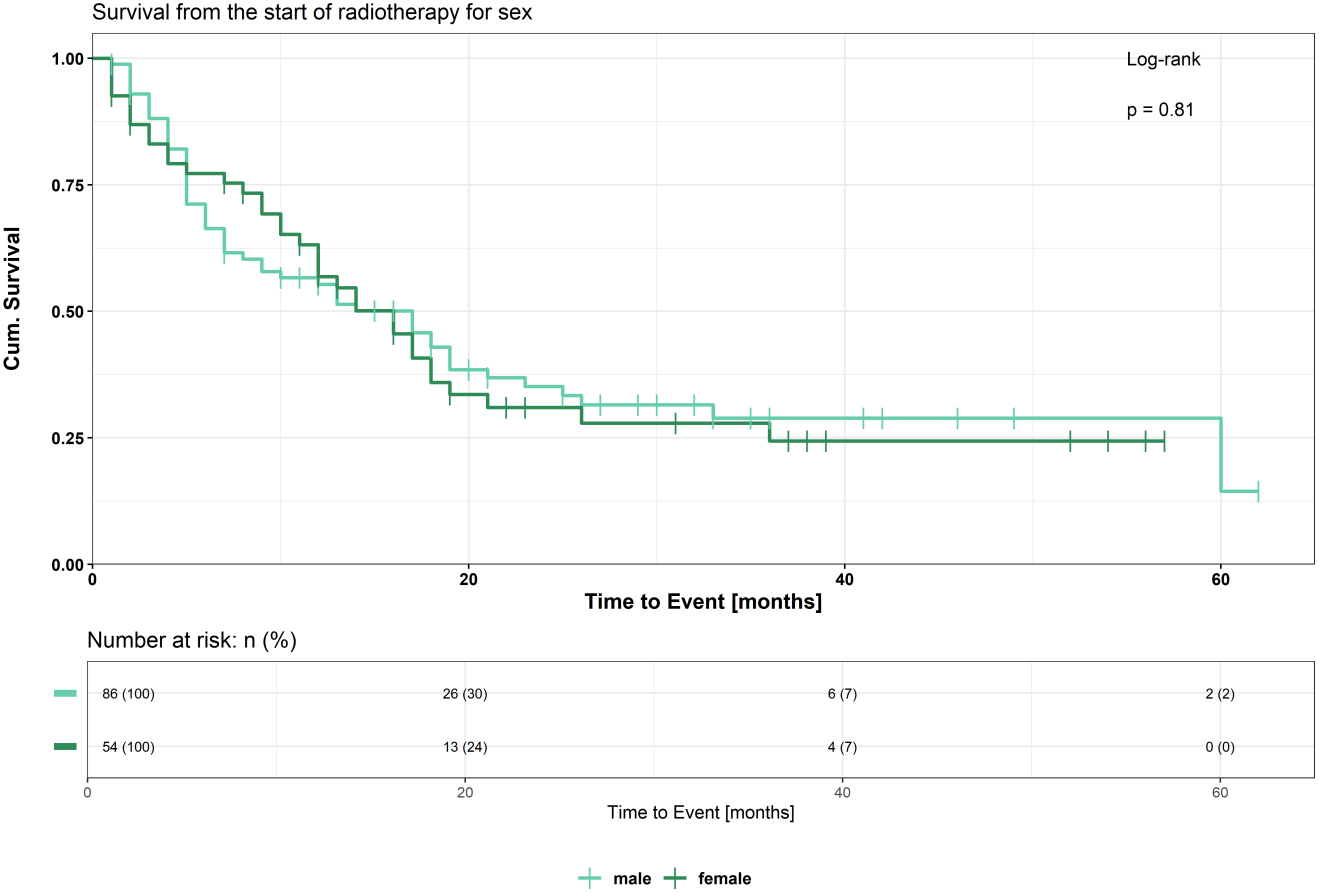
Kaplan-Meier curve for OS comparing male and female patients.

Kaplan-Meier survival curves of male and female patients are shown in **Figure 1**. Median OS was 16 months (95% CI: 12–21 months) for females and 17 months (95% CI: 9–21 months) for males. At 12 months, survival rates were 56.8% for females and 55.4% for males, while at 24 months, they were 31.0% and 35.1%, respectively. There was no significant difference in OS between men and women (p=0.81). 86 male and 54 female patients were included in this analysis. Similar results are shown for PFS, where we also did not see any significant differences between men and women (p=0.27) (**Supplementary Figure 3**).

The Cox regression analyses for n=29 female with 20 events (**Table 2**) and n=44 male with 27 events (**Table 3**) patients identified the following factors that were significant in the respective groups: For both females and males, a longer time interval between ICI and RT (in months) was associated with improved outcomes (female: p=0.002, HR = 0.748; male: p=0.012, HR = 0.903). Conversely, overweight (BMI ≥25 kg/m²) was negatively associated in both groups (female: p=0.028, HR = 4.801; male: p=0.008, HR = 4.282). A CRP value ≥5 mg/L was an unfavorable prognostic variable only in male patients (p=0.028, HR = 4.764). The low number of cases in the analysis, both for female and male patients, is due to missing data for independent variables. To assess the impact of missing CRP values, we performed a sensitivity analysis excluding this variable, which increased the number of included patients and events. The results for the remaining covariates were comparable, indicating that the main sex-specific associations were not driven by CRP inclusion.

**Table 2:** Cox regression for n=29 female patients with 20 events.

Term	Estimate (hazard ratio)	Std. error	Statistic	Confidence interval 95%	P-value
Age at diagnosis (years)	1	0.023	0.004	[0.956-1.047]	0.997
ICI end to RT start (months)	0.748	0.095	-3.069	[0.621 - 0.9]	**0.002**
RT localization: brain* vs. other	2.423	0.718	1.232	[0.593 - 9.897]	0.218
RT type: stereot.* vs. hypofr.	1.709	0.535	1.002	[0.599 - 4.877]	0.316
RT type: stereot.* vs. other	1.215	0.935	0.209	[0.195 - 7.59]	0.835
BMI <25 kg/m²* vs. ≥25 kg/m^2^	4.801	0.715	2.194	[1.182 - 19.502]	**0.028**
Prior RT: yes* vs. no	1.877	0.637	0.989	[0.539 - 6.539]	0.323
No/former smoker* vs. current Smoker	2.957	0.948	1.144	[0.461 - 18.967]	0.253
CRP <5 mg/L* vs. ≥5 mg/L	1.91	0.706	0.917	[0.479 - 7.615]	0.359

ICI, Immune Checkpoint Inhibitor; RT, Radiotherapy; BMI, body mass index; CRP, C-reactive protein; stereot, (stereotactic), ultrahypofractionated/stereotactic, hypofr., hypofractionated, *reference. significant values in bold.

**Table 3:** Cox regression for n=44 male patients with 27 events.

Term	Estimate (hazard ratio)	Std. error	Statistic	Confidence interval 95%	P-value
Age at diagnosis (years)	0.988	0.025	-0.483	[0.941 - 1.038]	0.629
ICI end to RT start (months)	0.903	0.041	-2.5	[0.833 - 0.978]	**0.012**
RT localization: brain* vs. other	0.793	0.555	-0.417	[0.267 - 2.356]	0.677
RT type: stereot.* vs. hypofr.	1.506	0.664	0.616	[0.41 - 5.533]	0.538
RT type: stereot.* vs. other	1.772	0.812	0.704	[0.361 - 8.71]	0.481
BMI <25 kg/m²* vs. ≥25 kg/m^2^	4.282	0.544	2.672	[1.473 - 12.446]	**0.008**
Prior RT: yes* vs. no	0.86	0.51	-0.296	[0.316 - 2.337]	0.767
No/former smoker* vs. current Smoker	0.524	0.614	-1,052	[0.157 - 1.746]	0.293
CRP <5 mg/L* vs. ≥5 mg/L	4.764	0.71	2.198	[1.184 - 19.17]	**0.028**

ICI, Immune Checkpoint Inhibitor; RT, Radiotherapy; BMI, body mass index; CRP, C-reactive protein, stereot. (stereotactic), ultrahypofractionated/stereotactic, hypofr., hypofractionated. *reference. significant values in bold.

**Figure 2:**
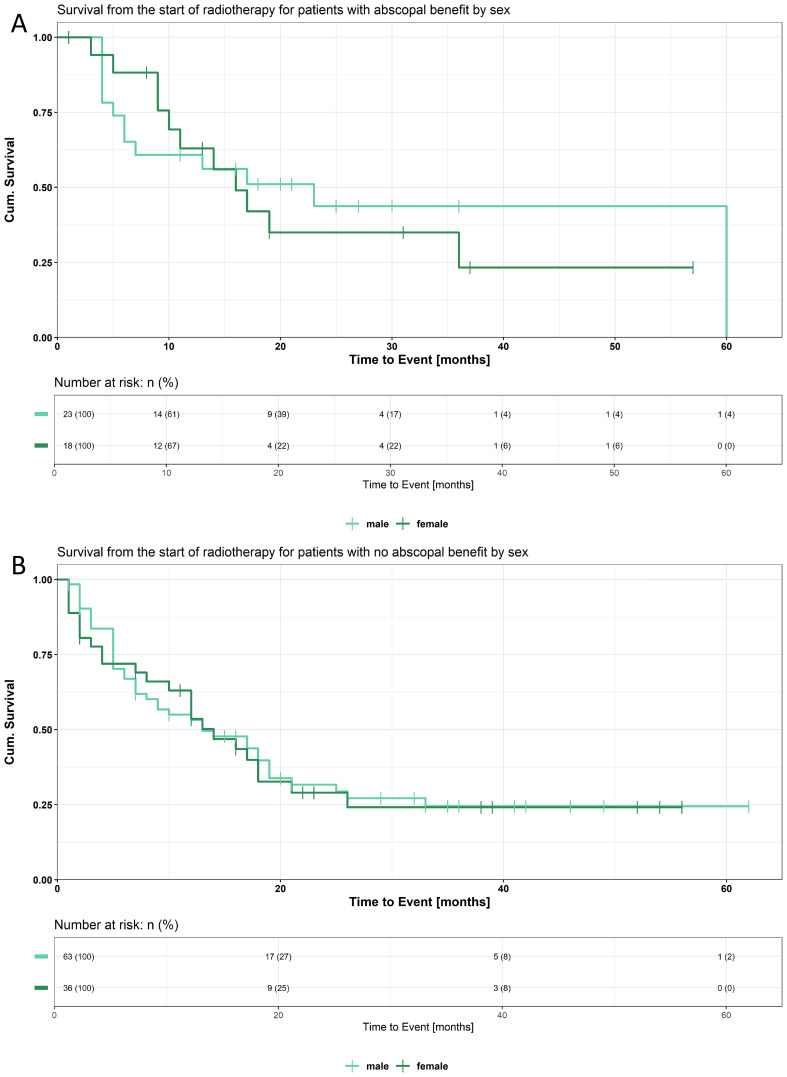
**(A)** Kaplan-Meier curve for OS comparing male and female patients with abscopal benefit. **(B)** Kaplan-Meier curve for OS comparing male and female patients without abscopal benefit.

Kaplan-Meier survival curves for patients with AB (A) and no AB (B) with respect to sex are demonstrated in **Figure 2**. The median OS with AB was 16 months for females and 23 months for males. The 12-month survival rates were 63.0% for females and 60.9% for males, decreasing to 35.0% and 43.8%, respectively, at 24 months. A total of 23 male and 18 female patients were included in the analysis. The median OS with no AB was 14 months (95% CI: 10–26 months) for females and 13 months (95% CI: 8–19 months) for males. Survival at 12 months was 53.6% for females and 53.3% for males, while at 24 months, it was 29.0% and 31.7%, respectively. This analysis included 63 male and 36 female patients. The median PFS with AB was 9 months for females and 6 months for males. The 12-month survival rates were 30.9% for females and 39.1% for males, decreasing to 15.4% and 24.5%, respectively, at 24 months. A total of 23 male and 18 female patients were included in the analysis. The median PFS with no AB was 4 months (95% CI: 2–6 months) for females and 5 months (95% CI: 4–7 months) for males. Survival at 12 months was 18.3% for females and 27.3% for males, while at 24 months, it was 9.2% and 19.3%, respectively. This analysis included 62 male and 36 female patients. The results for PFS are shown in **Supplementary Figure 4**.

We further analyzed survival with respect to sex in the different response groups separately (see **Supplementary Figure 5**). The median OS for patients with AR was 15 months (95% CI: 10–57 mo.) for females, while it was not reached for male patients. At 12 months, survival was 58.3% for females and 66.7% for males, and at 24 months, 25.0% and 53.3%, respectively. A total of 12 male and 13 female patients were analyzed (**Supplementary Figure 5A**). The median OS for patients with ≥1AR was 13 months (95% CI: 4–36 mo.) in males. Survival at 12 months was 75.0% for females and 54.5% for males, while at 24 months, it was 75.0% and 29.1%, respectively. This analysis included 11 male and 5 female patients (**Supplementary Figure 5B**). Median OS for patients with AC was 18 months (95% CI: 12–56 mo.) for females and 18 months (95% CI: 17–62 mo.) for males. At 12 months, survival rates were 66.8% for females and 71.6% for males, while at 24 months, they were 42.5% and 35.8%, respectively. 26 male and 19 female patients were included in this analysis (**Supplementary Figure 5C**). Similar results are shown for PFS (**Supplementary Figure 6**): The median PFS for patients with AR was 7 months for females and 8 months for male patients. At 12 months, survival was 23.1% for females and 41.7% for males, and at 24 months, 7.7% and 25.0%, respectively. A total of 12 male and 13 female patients were analyzed (**Supplementary Figure 6A**). The median PFS for patients with ≥1AR was not reached for females and in males it was 4 months. Survival at 12 months was 53.3% for females and 36.4% for males. At 24 months, it remained at 53.3% for females and was 24.2% for males. This analysis included 11 male and 5 female patients (**Supplementary Figure 6B**). Median PFS for patients with AC was 4 months (95% CI: 3–17 mo.) for females and 7 months (95% CI: 5–26 mo.) for males. At 12 months, survival rates were 30.1% for females and 43.7% for males, while at 24 months, they were 18.0% and 35%, respectively. 26 male and 19 female patients were included in this analysis (**Supplementary Figure 6C**). An analysis for patients with AP could not be conducted as there was only one female patient included.

## Discussion

The ARTIC study reveals several critical insights into sex-specific patterns of AbE and treatment response following combined radiotherapy (RT) and immune checkpoint inhibition (ICI). Our findings demonstrated a notable difference in AR and AC rates between males and females, with females showing higher rates of both (AR: 24% vs. 14%; AC: 35% vs. 31%). Notably, there was also only one female patient with AP. These observations align with emerging evidence of sex-based immunological variations.

Sex-based differences in immune responses are known to be attributed to multiple factors, including the effects of sex hormones on immune cell function, X-chromosome-linked immune regulatory genes, and variations in microbiome (17, 18). Estrogen, in particular, has been shown to enhance T-cell activation, promote pro-inflammatory cytokine production, and influence macrophage polarization - all critical components in the immune response to RT and ICI (19, 20). Xiao et al. (21) provide a comprehensive mechanistic framework for these differences in their 2024 review. They delineate how sex chromosomes and hormones fundamentally modulate immune responses, with females exhibiting enhanced type I interferon responses, more robust T-cell activation mechanisms, and differential gene expression in immune-related pathways - observations that add nuance to our findings of higher AR and AC rates in females. Interestingly, the review highlights that while female individuals generally experience more severe adverse events to immune checkpoint blockade, male individuals often show more favorable responses to such treatments, which has also been shown in reviews by Conforti et al. (9, 10).

The optimal sequence and timing interval between ICI and RT is currently an area of intensive research (22). Our sex-stratified analysis revealed significant results regarding treatment timing. The interval between ICI cessation and RT initiation showed distinct prognostic implications for males (HR = 0.903, p=0.012) as well as females (HR = 0.748, p=0.002). Longer intervals were an independent prognostic marker for longer OS. The effect was more pronounced in female patients. A longer response to ICI therapy may contribute to more prolonged OS, as these patients may not require radiotherapy for symptomatic disease early on. However, the retrospective nature of our study presents a limitation in drawing definitive conclusions.

In our analysis, an overweight BMI of ≥25 kg/m² was an independent prognostic marker for both male (HR = 4.282, p=0.008) and female (HR = 4.801, p=0.028) patients, with females exhibiting a slightly higher mortality risk compared to males. This is in line with evidence from Ihara et al. (23) who demonstrated a reduced response to ICI in patients with NSCLC and overweight. On the other hand, McQuade et al. (24) reported that an elevated BMI was associated with better treatment outcomes under targeted therapy, as well as immunotherapy and chemotherapy. Interestingly, this effect was especially pronounced in male patients, which may point to sex-specific differences in immunometabolism. Vedire et al. (25) further elaborate on the interaction between sex and obesity on the tumor microenvironment in NSCLC, suggesting that metabolic factors interact differently with immune system activation in males and females. Their data demonstrated that tumor inflammation and PD-L1 expression are more strongly associated with BMI in women than in men.

The finding that elevated CRP levels (≥5 mg/L) were prognostically significant exclusively in males (HR = 4.764, p=0.028) is an interesting observation. Clinical and epidemiological studies have suggested a strong association among chronic infection, chronic inflammation, and cancer. The role of systemic inflammation in modulating treatment response has gained increasing attention, with CRP emerging as a promising biomarker (26). Although elevated chronic inflammation levels are generally associated with poorer outcomes in various malignancies, particularly NSCLC (27, 28), growing evidence suggests that acute inflammatory signaling may, under certain conditions, enhance abscopal responses - especially when combined with RT-induced antigen release and checkpoint blockade. Its impact therefore appears to be highly dependent on both the context and the timing of RT–ICI administration (29). Reports and reviews describe AbE occurring alongside inflammatory activation, underscoring this bidirectional biology (30, 31). Recent studies suggest sex-specific differences in its prognostic value (25, 32). These differences may reflect underlying variations in inflammatory pathway activation and immune system regulation between males and females (33). Recent research suggests that inflammatory responses are fundamentally modulated by sex-specific genetic and hormonal factors (21). This aligns perfectly with our observation of sex-specific CRP significance, indicating that inflammatory biomarkers may require nuanced, sex-specific interpretation.

Our findings resonate with the emerging paradigm highlighted in recent publications that sex represents a crucial biological variable in cancer immunotherapy research that has been historically underappreciated. These findings are supported by emerging clinical data: Petersen et al. (34) observed sex-specific patterns in treatment outcomes among melanoma patients receiving immune checkpoint inhibitors in a large Danish cohort. Their study demonstrated that female patients showed better treatment outcomes compared to males, with sex being identified as an independent predictive variable. Mo et al. (35) reported sex-specific differences for small-cell lung cancer from immunotherapy advancement, with significant benefits especially for female patients.

In contrast, Cohen et al. (36) found no significant sex-related differences in PFS and OS in NSCLC patients. However, female patients had a significantly higher risk of severe immune-mediated adverse events (irAEs) leading to treatment discontinuation. Particularly, gastrointestinal toxicities such as hepatitis and colitis occurred more frequently in women, while pneumonitis was the most common irAE in men. These recent findings collectively underscore the growing recognition that sex represents a crucial biological variable in determining immunotherapy efficacy, suggesting that sex-specific considerations should be integrated into treatment planning and response assessment.

Our cohort largely reflects patients already exposed to ICIs, where progression on therapy suggests secondary (acquired) resistance in a substantial fraction. Consensus definitions from the Society for Immunotherapy of Cancer formalize this clinical state and explicitly motivate strategies to restore sensitivity (37). In this context, focal RT may re-prime antitumor immunity by increasing antigen release/presentation and type-I interferon signaling, thereby enabling continuation or re-challenge of ICI beyond radiographic progression in selected patients. Clinical series and reviews report that adding hypofractionated RT in the setting of ICI resistance can achieve meaningful disease control and allow ongoing ICI in many cases (38). These observations offer a mechanistic and translational framework for our lesion-level findings and support prospective testing of RT as a means to mitigate secondary ICI resistance.

Understanding these sex-specific patterns could be crucial for optimizing patient selection and treatment timing in combined RT-ICI approaches. Potential future research directions could include developing sex-specific biomarker panels, creating gender-stratified treatment algorithms, and investigating the molecular mechanisms underlying these observed differences. Future work should prospectively test strategies to increase AR likelihood: individually defined RT-ICI dose, fractionation and sequencing, biomarker-guided and sex-stratified strategies, but also specifications which lesions are the best to treat for a most personal and precise immunoradiotherapy.”

## Limitations

While our study provides valuable insights and supports recent findings of other authors, we acknowledge limitations. Due to the retrospective design and this being a secondary analysis, the sample size for some of the analysis was relatively small, primarily as a result of missing values. This limitation, compounded by the cohort’s overall heterogeneity, reduces the statistical power and increases the risk of false-negative or false-positive findings. Per-lesion dosimetry for NIL was not retained in the anonymized multi-center export, so we cannot definitively exclude low-dose exposure below the 10% isodose line for all NIL. Future prospective studies should incorporate uniform per-lesion DVHs and dose-thresholds for NIL definitions to directly quantify and minimize potential low-dose spill. Also, unmeasured confounders such as PD-L1, sex hormones, or concomitant medications limit causal inference. Incomplete laboratory documentation represents a limitation and highlights the need for standardized data collection in future research. With regards to our sex-associated CRP findings, it is important to consider that missing data in this study were handled by complete-case analysis, as the use of multiple imputation or weighting methods was considered inappropriate given the exploratory design and potential for non-random missingness. In particular, CRP values were missing in approximately 30% of patients. Since CRP was a significant prognostic factor in males but not in females, we conducted a sensitivity analysis excluding this variable, which increased the sample size and confirmed that the main associations for other covariates remained consistent. Pooled Cox models containing interaction terms between sex and other covariates could provide further insights, but our study design and the available event numbers did not allow for a reliable estimation of such complex interactions. Therefore, separate sex-specific analyses were performed. Future prospective research should also include different timing models analyzing also RT as first treatment, followed by ICI. Finally, there is a notable risk of sampling bias, and our data are limited to specific cancer types and treatment protocols. All results should be interpreted as exploratory.

## Conclusion

The ARTIC study provides evidence for sex-associated variations in AbE of combined RT and ICI, as well as immune response. Our findings align with and extend recent literature, emphasizing the critical importance of considering sex as a fundamental biological variable in cancer treatment strategies.

## Data Availability

The raw data supporting the conclusions of this article will be made available by the authors when requested, without undue reservation.

## Supplementary material

Supplementary Figure 1 Flow Chart showing the conduct of the study. RT = radiotherapy, ICI = immune checkpoint inhibitors, PD-L1 = programmed cell death ligand-1, PD-1 = programmed cell death protein 1, CTLA-4 = cytotoxic T-lymphocyte-associated protein 4, CR = complete response, PR = partial response, VMAT = volumetric intensity modulated arc therapy, IMRT = intensity-modulated radiation therapy, OS = overall survival, PFS = progression-free survival

Supplementary Figure 2 Stratification of NILs (non-irradiated lesions) and patients for abscopal effect, further summarized in abscopal benefit or no abscopal benefit groups. AR = Abscopal Response, AC = Abscopal Control, AP = Abscopal Progression, MR = Mixed Response

Supplementary Figure 3 Kaplan-Meier curve for PFS comparing male and female patients

Supplementary Figure 4 **(A)** Kaplan-Meier curve for PFS comparing male and female patients with abscopal benefit; **(B)**: Kaplan-Meier curve for PFS comparing male and female patients without abscopal benefit

Supplementary Figure 5 **(A)** Kaplan-Meier curve for OS comparing male and female patients within the abscopal response (AR) group; **(B)**: Kaplan-Meier curve for OS comparing male and female patients within the ≥1 AR group; **(C)**: Kaplan-Meier curve for OS comparing male and female patients within the abscopal control (AC) group

Supplementary Figure 6 **(A)** Kaplan-Meier curve for PFS comparing male and female patients within the abscopal response (AR) group; **(B)**: Kaplan-Meier curve for PFS comparing male and female patients within the ≥1 AR group; **(C)**: Kaplan-Meier curve for PFS comparing male and female patients within the abscopal control (AC) group
