# Supplementary material for: Sex-specific differences in abscopal responses to combined radiotherapy and immune checkpoint inhibition–insights from a multicenter study
Source: Front Immunol. 2026 Feb 2;16:1699362. doi: 10.3389/fimmu.2025.1699362 (PMC12907404; doi:10.3389/fimmu.2025.1699362)
Supplement: Supplementary Figure 1 — Flow Chart showing the conduct of the study. RT = radiotherapy, ICI = immune checkpoint inhibitors, PD-L1 = programmed cell death ligand-1, PD-1 = programmed cell death protein 1, CTLA-4 = cytotoxic T-lymphocyte-associated protein 4, CR = complete response, PR = partial response, VMAT = volumetric intensity modulated arc therapy, IMRT = intensity-modulated radiation therapy, OS = overall survival, PFS = progression-free survival [file Image1.pdf]

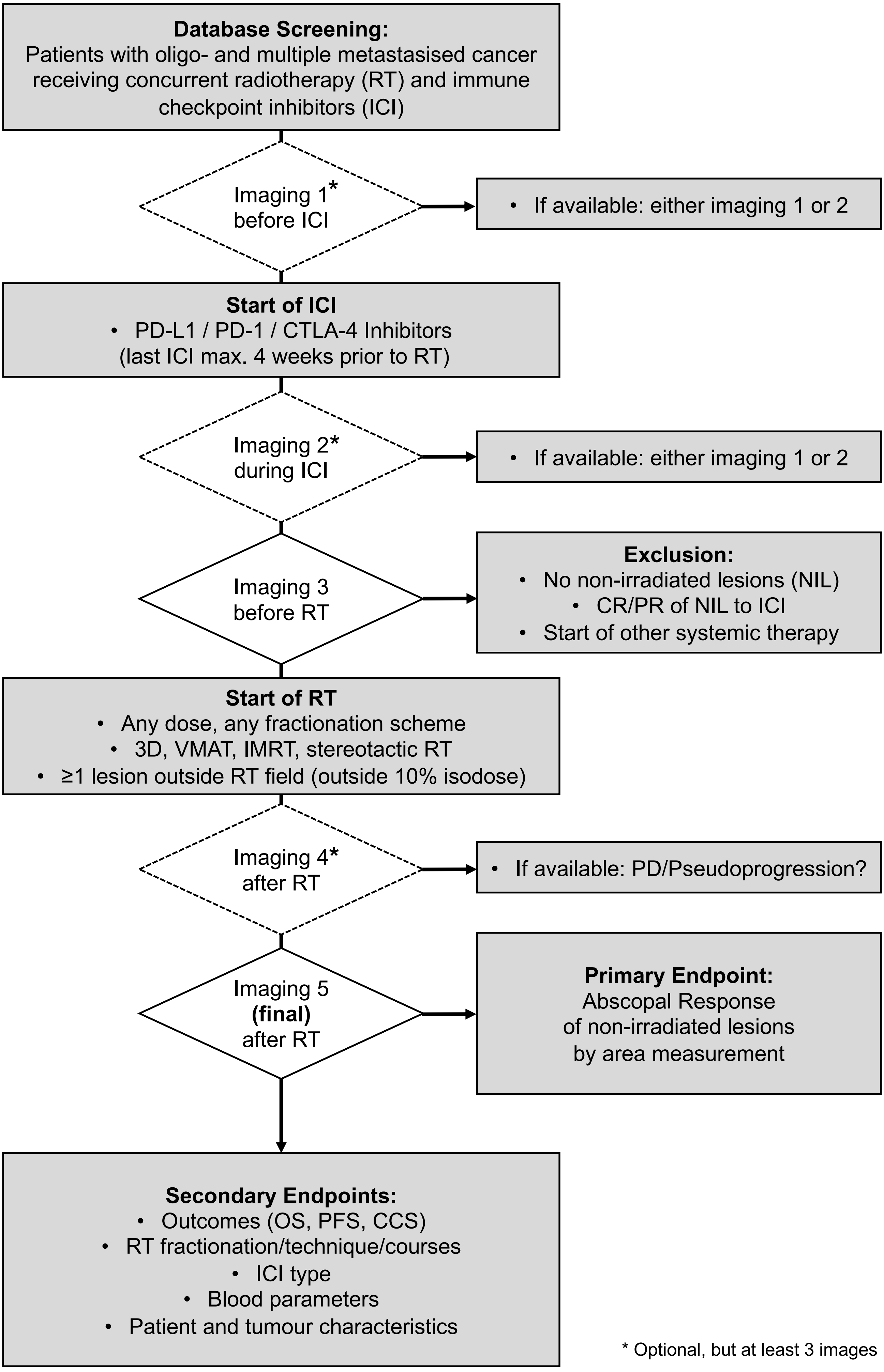

Database Screening:
Patients with oligo- and multiple metastasised cancer receiving concurrent radiotherapy (RT) and immune checkpoint inhibitors (ICI)
Imaging 1* before ICI
• If available: either imaging 1 or 2
Start of ICI
• PD-L1 / PD-1 / CTLA-4 Inhibitors (last ICI max. 4 weeks prior to RT)
Imaging 2* during ICI
• If available: either imaging 1 or 2
Imaging 3 before RT
Exclusion:
• No non-irradiated lesions (NIL)
• CR/PR of NIL to ICI
• Start of other systemic therapy
Start of RT
• Any dose, any fractionation scheme
• 3D, VMAT, IMRT, stereotactic RT
• ≥1 lesion outside RT field (outside 10% isodose)
Imaging 4* after RT
• If available: PD/Pseudoprogression?
Imaging 5 (final) after RT
Primary Endpoint:
Abscopal Response of non-irradiated lesions by area measurement
Secondary Endpoints:
• Outcomes (OS, PFS, CCS)
• RT fractionation/technique/courses
• ICI type
• Blood parameters
• Patient and tumour characteristics
* Optional, but at least 3 images
