# Supplementary material for: Sex-specific differences in abscopal responses to combined radiotherapy and immune checkpoint inhibition–insights from a multicenter study
Source: Front Immunol. 2026 Feb 2;16:1699362. doi: 10.3389/fimmu.2025.1699362 (PMC12907404; doi:10.3389/fimmu.2025.1699362)
Supplement: Supplementary Figure 2 — Stratification of NILs (non-irradiated lesions) and patients for abscopal effect, further summarized in abscopal benefit or no abscopal benefit groups. AR = Abscopal Response, AC = Abscopal Control, AP = Abscopal Progression, MR = Mixed Response [file Image2.pdf]

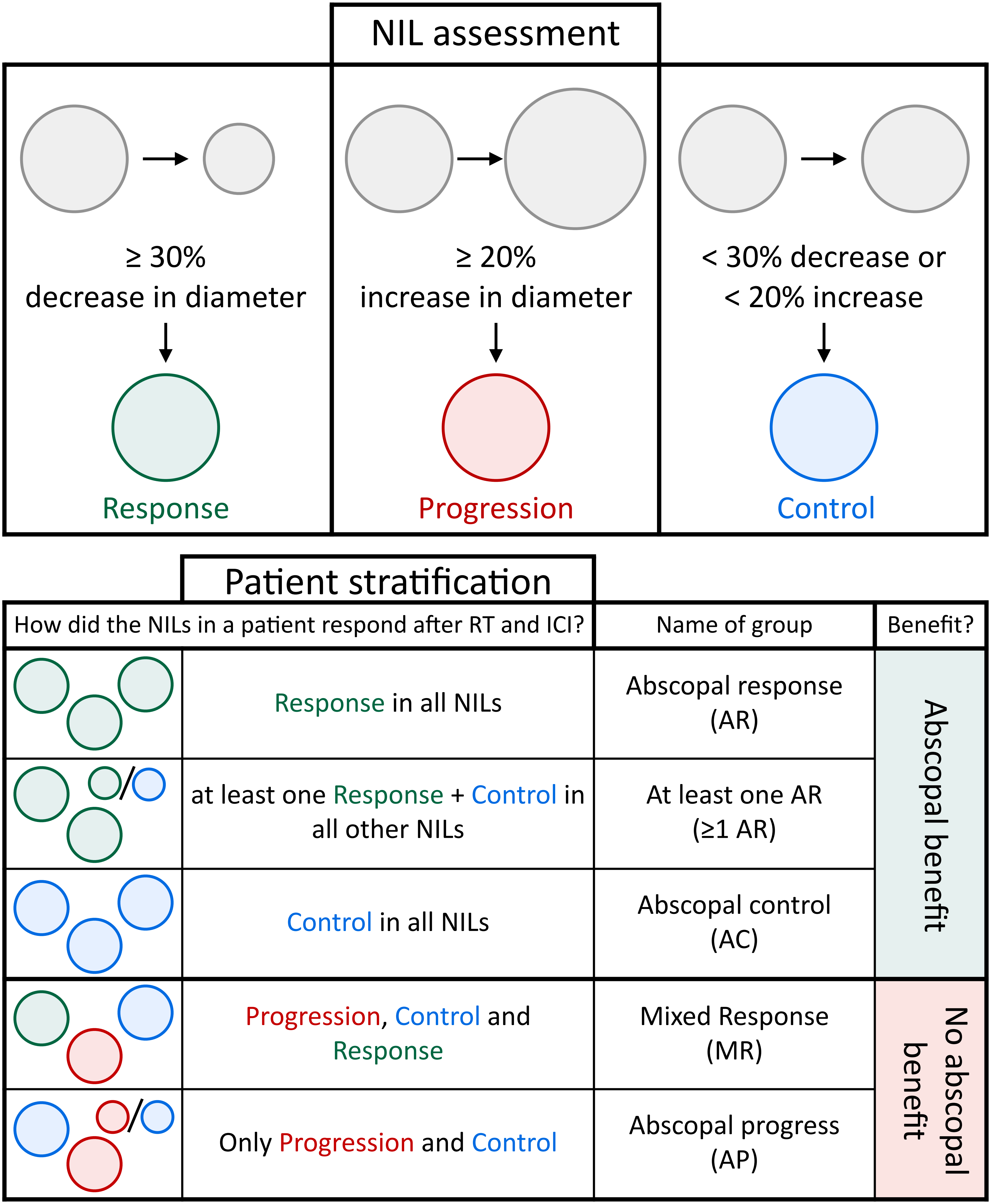

NIL assessment
≥ 30%
decrease in diameter
Response
≥ 20%
increase in diameter
Progression
< 30% decrease or
< 20% increase
Control
Patient stratification
How did the NILs in a patient respond after RT and ICI?
Name of group
Benefit?
Response in all NILs
Abscopal response (AR)
at least one Response + Control in all other NILs
At least one AR (≥1 AR)
Control in all NILs
Abscopal control (AC)
Abscopal benefit
Progression, Control and Response
Mixed Response (MR)
Only Progression and Control
Abscopal progress (AP)
No abscopal benefit
