# Supplementary figures and images for: Sex-specific differences in abscopal responses to combined radiotherapy and immune checkpoint inhibition–insights from a multicenter study
Source: Front Immunol. 2026 Feb 2;16:1699362. doi: 10.3389/fimmu.2025.1699362 (PMC12907404; doi:10.3389/fimmu.2025.1699362)

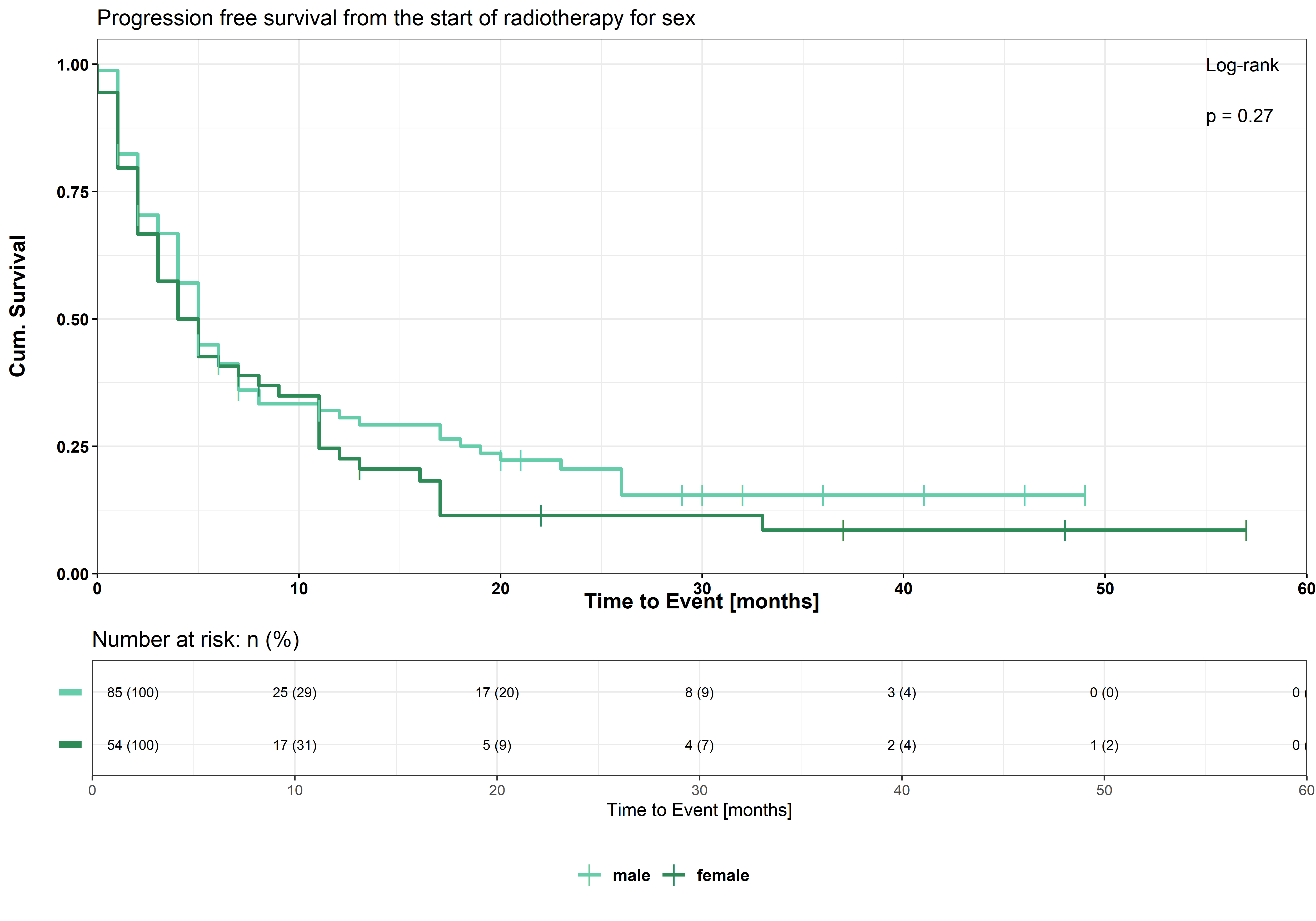

Supplement: Supplementary Figure 3 — Kaplan-Meier curve for PFS comparing male and female patients [file Image3.tiff]

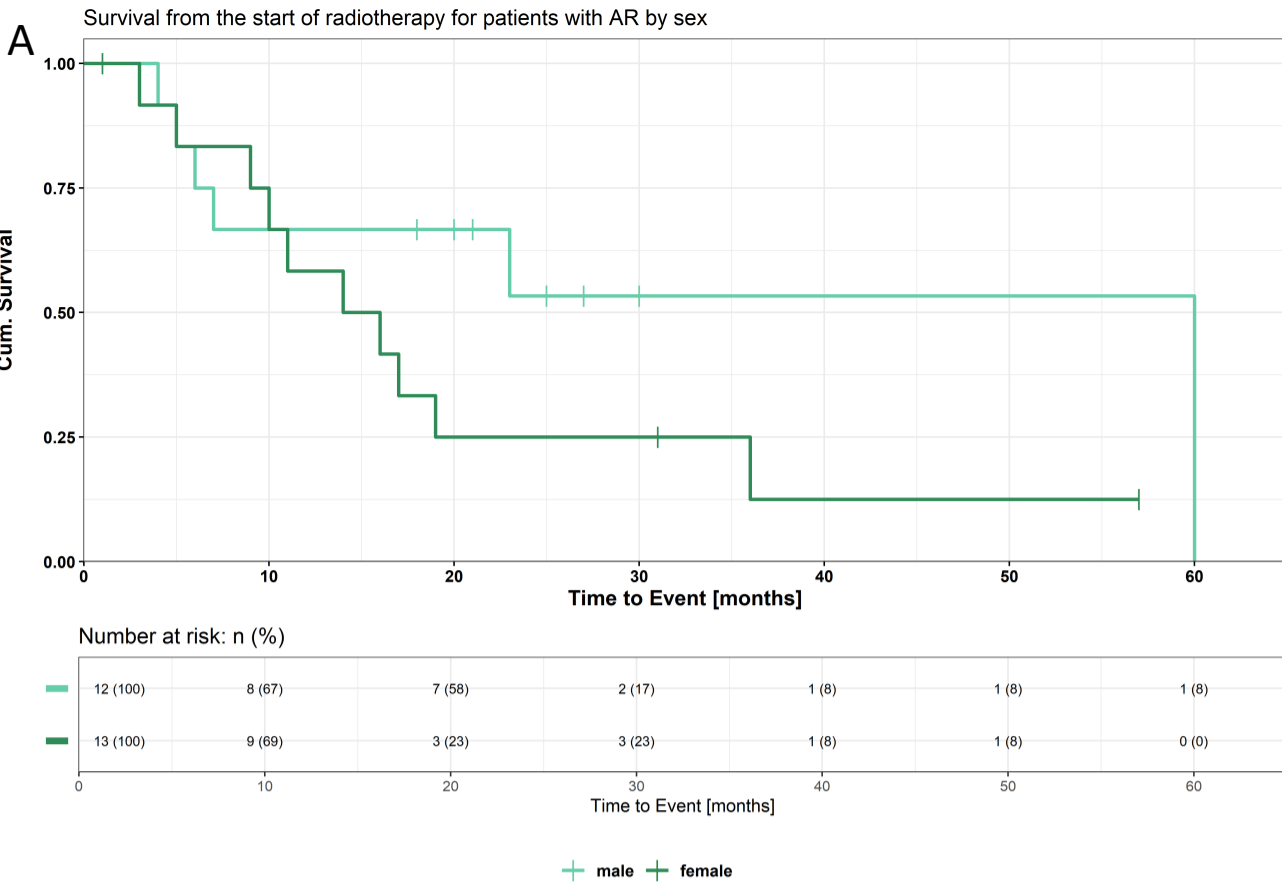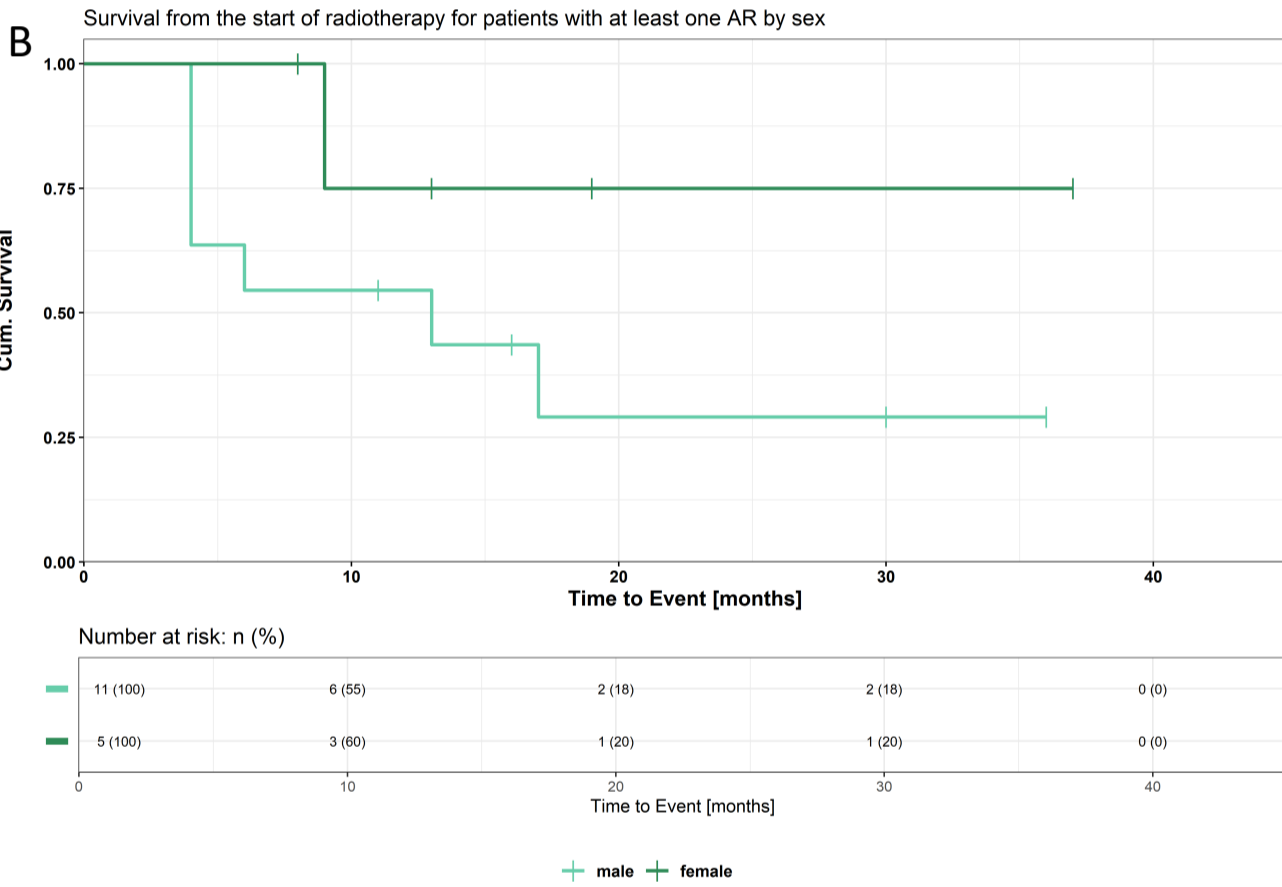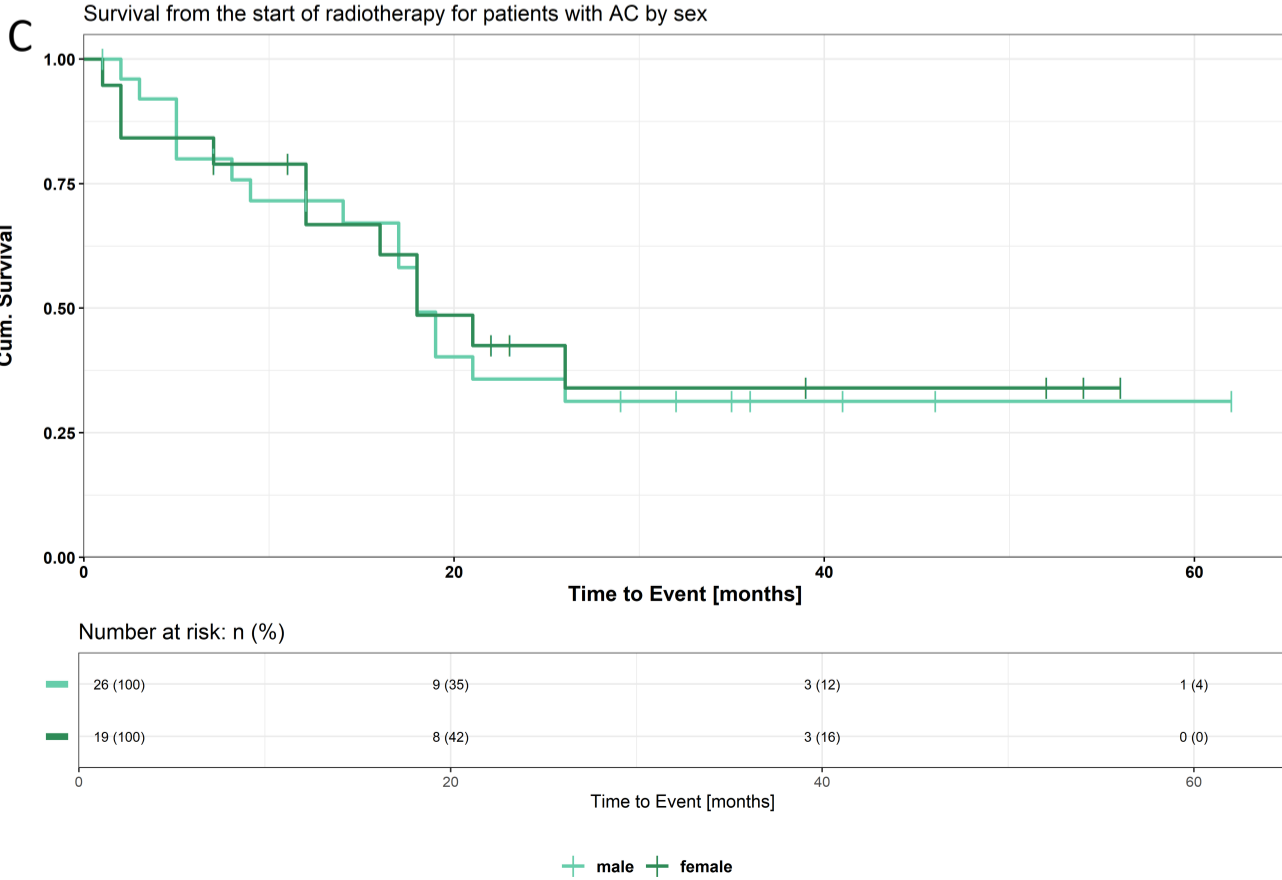

Supplement: Supplementary Figure 5 — (A) Kaplan-Meier curve for OS comparing male and female patients within the abscopal response (AR) group; (B): Kaplan-Meier curve for OS comparing male and female patients within the ≥1 AR group; (C): Kaplan-Meier curve for OS comparing male and female patients within the abscopal control (AC) group [file Image5.pdf]
