# Supplementary material for: Sex-specific differences in abscopal responses to combined radiotherapy and immune checkpoint inhibition–insights from a multicenter study
Source: Front Immunol. 2026 Feb 2;16:1699362. doi: 10.3389/fimmu.2025.1699362 (PMC12907404; doi:10.3389/fimmu.2025.1699362)
Supplement: Supplementary Figure 4 — (A) Kaplan-Meier curve for PFS comparing male and female patients with abscopal benefit; (B): Kaplan-Meier curve for PFS comparing male and female patients without abscopal benefit [file Image4.pdf]

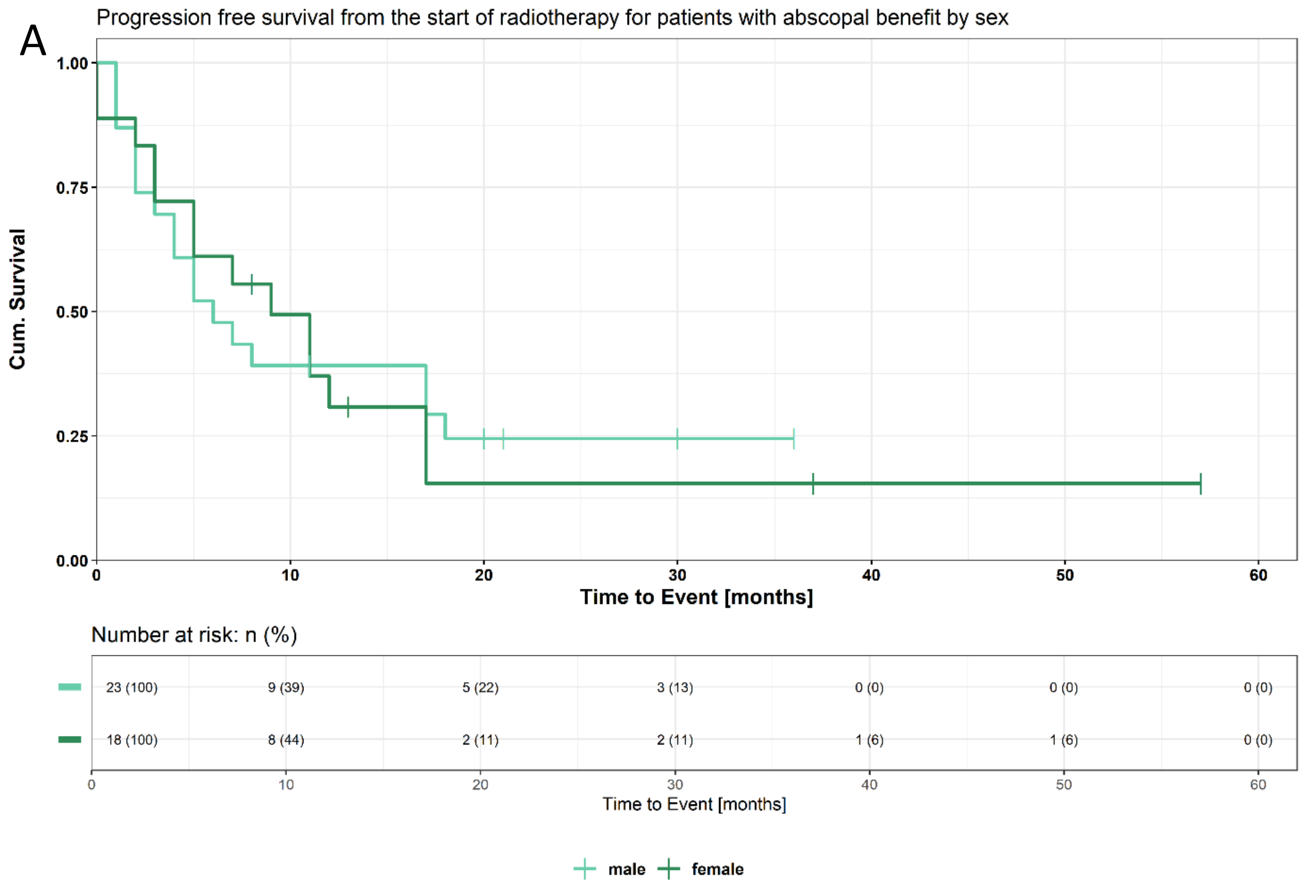
A
Progression free survival from the start of radiotherapy for patients with abscopal benefit by sex
Cum. Survival
1.00
0.75
0.50
0.25
0.00
0 10 20 30 40 50 60
Time to Event [months]
Number at risk: n (%)
23 (100) 9 (39) 5 (22) 3 (13) 0 (0) 0 (0) 0 (0)
18 (100) 8 (44) 2 (11) 2 (11) 1 (6) 1 (6) 0 (0)
0 10 20 30 40 50 60
Time to Event [months]
male
female

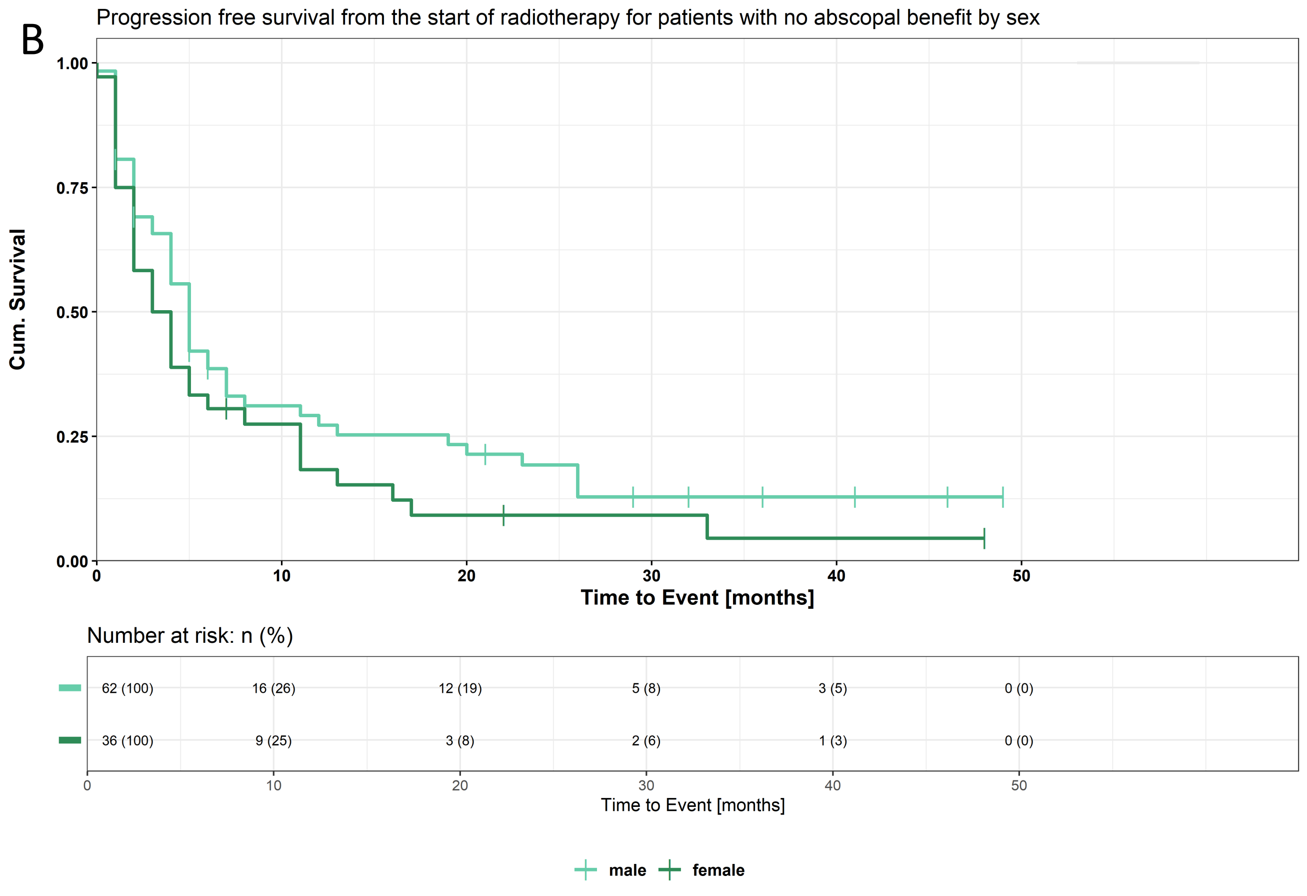
B
Progression free survival from the start of radiotherapy for patients with no abscopal benefit by sex
Cum. Survival
1.00
0.75
0.50
0.25
0.00
0 10 20 30 40 50
Time to Event [months]
Number at risk: n (%)
62 (100) 16 (26) 12 (19) 5 (8) 3 (5) 0 (0)
36 (100) 9 (25) 3 (8) 2 (6) 1 (3) 0 (0)
0 10 20 30 40 50
Time to Event [months]
male
female
