# Supplementary material for: Sex-specific differences in abscopal responses to combined radiotherapy and immune checkpoint inhibition–insights from a multicenter study
Source: Front Immunol. 2026 Feb 2;16:1699362. doi: 10.3389/fimmu.2025.1699362 (PMC12907404; doi:10.3389/fimmu.2025.1699362)
Supplement: Supplementary Figure 6 — (A) Kaplan-Meier curve for PFS comparing male and female patients within the abscopal response (AR) group; (B): Kaplan-Meier curve for PFS comparing male and female patients within the ≥1 AR group; (C): Kaplan-Meier curve for PFS comparing male and female patients within the abscopal control (AC) group [file Image6.pdf]

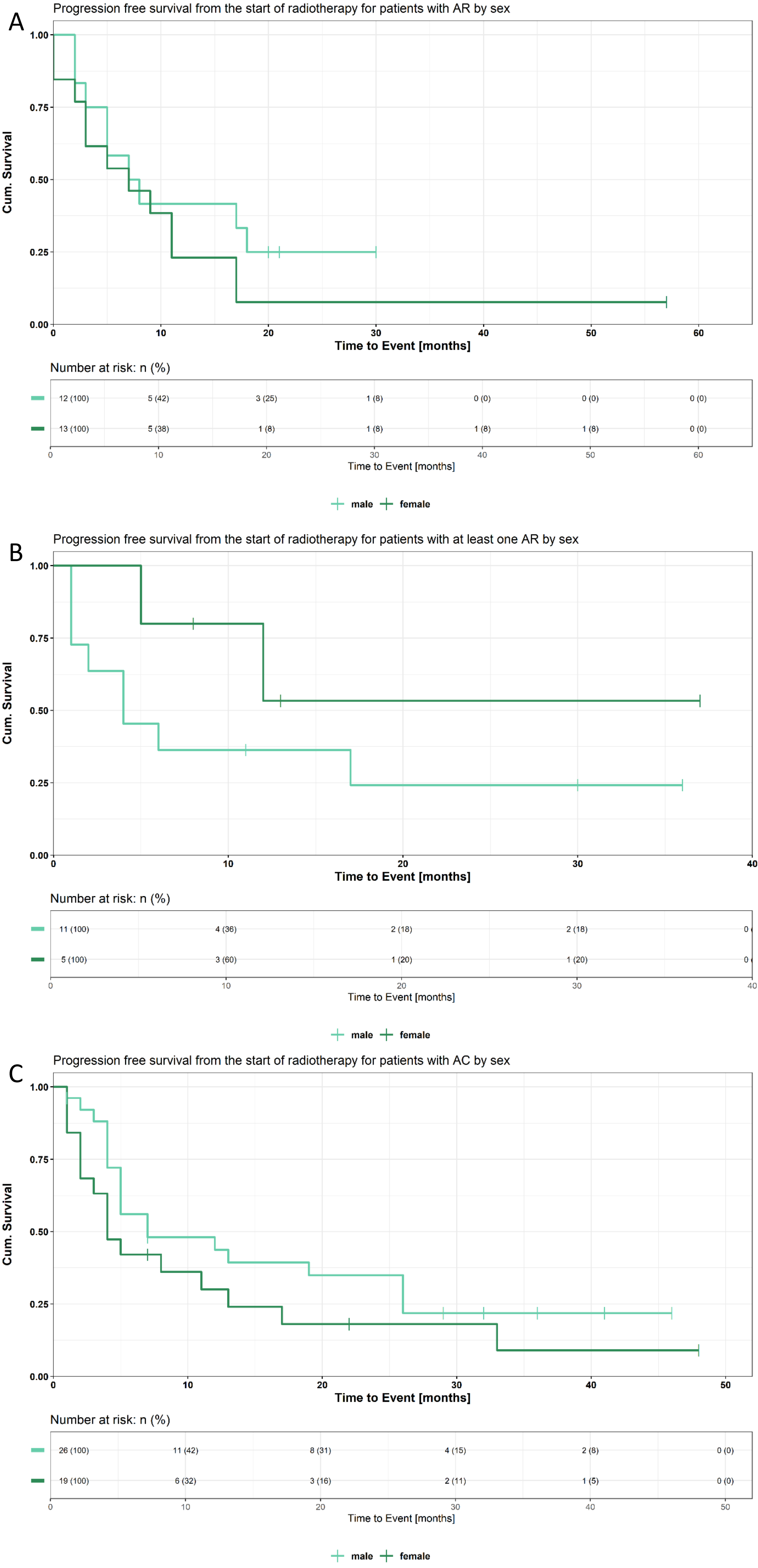
A
Progression free survival from the start of radiotherapy for patients with AR by sex
Cum. Survival
1.00
0.75
0.50
0.25
0.00
0
10
20
30
40
50
60
Time to Event [months]
Number at risk: n (%)
12 (100) 5 (42) 3 (25) 1 (8) 0 (0) 0 (0) 0 (0)
13 (100) 5 (38) 1 (8) 1 (8) 1 (8) 1 (8) 0 (0)
Time to Event [months]
male
female
B
Progression free survival from the start of radiotherapy for patients with at least one AR by sex
Cum. Survival
1.00
0.75
0.50
0.25
0.00
0
10
20
30
40
Time to Event [months]
Number at risk: n (%)
11 (100) 4 (36) 2 (18) 2 (18) 0
5 (100) 3 (60) 1 (20) 1 (20) 0
Time to Event [months]
male
female
C
Progression free survival from the start of radiotherapy for patients with AC by sex
Cum. Survival
1.00
0.75
0.50
0.25
0.00
0
10
20
30
40
50
Time to Event [months]
Number at risk: n (%)
26 (100) 11 (42) 8 (31) 4 (15) 2 (8) 0 (0)
19 (100) 6 (32) 3 (16) 2 (11) 1 (5) 0 (0)
Time to Event [months]
male
female
